# Interplay Between Iron Overload and Osteoarthritis: Clinical Significance and Cellular Mechanisms

**DOI:** 10.3389/fcell.2021.817104

**Published:** 2022-01-14

**Authors:** Chenhui Cai, Wenhui Hu, Tongwei Chu

**Affiliations:** ^1^ Department of Orthopedics, Xinqiao Hospital, Third Military Medical University (Army Medical University), Chongqing, China; ^2^ Department of Biomedical Materials Science, Third Military Medical University (Army Medical University), Chongqing, China

**Keywords:** osteoarthritis, iron overload, cartilage, subchondral bone, synovium

## Abstract

There are multiple diseases or conditions such as hereditary hemochromatosis, hemophilia, thalassemia, sickle cell disease, aging, and estrogen deficiency that can cause iron overload in the human body. These diseases or conditions are frequently associated with osteoarthritic phenotypes, such as progressive cartilage degradation, alterations in the microarchitecture and biomechanics of the subchondral bone, persistent joint inflammation, proliferative synovitis, and synovial pannus. Growing evidences suggest that the conditions of pathological iron overload are associated with these osteoarthritic phenotypes. Osteoarthritis (OA) is an important complication in patients suffering from iron overload-related diseases and conditions. This review aims to summarize the findings and observations made in the field of iron overload-related OA while conducting clinical and basic research works. OA is a whole-joint disease that affects the articular cartilage lining surfaces of bones, subchondral bones, and synovial tissues in the joint cavity. Chondrocytes, osteoclasts, osteoblasts, and synovial-derived cells are involved in the disease. In this review, we will elucidate the cellular and molecular mechanisms associated with iron overload and the negative influence that iron overload has on joint homeostasis. The promising value of interrupting the pathologic effects of iron overload is also well discussed for the development of improved therapeutics that can be used in the field of OA.

## 1 Introduction

Osteoarthritis (OA) is a common degenerative and progressive joint disease that is the primary cause of joint pain, joint dysfunction and deformity, and limb disability. This disease negatively affects the quality of life. (Hunter et al., 2020). The term OA is used to define inflammatory diseases that occur in the joint and surrounding tissues of the human body and are caused by joint biomechanics, degeneration, trauma, or other multiple metabolic factors (Xia et al., 2014). However, complicated mechanisms involved in the pathogenesis of OA significantly limit the development of biological disease-modifying OA drugs (Van Spil et al., 2019).

Studies reported in the past decades have revealed the association between iron homeostasis and OA. Diseases or conditions with diverse etiologies can result in iron overload (Jeney, 2017). Iron overload is also associated with OA of the joints in patients suffering from diseases associated with iron overload (such as hereditary hemochromatosis (HH), thalassemia, hemophilia, sickle cell disease (SCD)). Iron overload can also result in aging and estrogen deficiency (Dallos et al., 2013; Chehade and Adams, 2019; Simão and Cancela, 2021). HH is the leading case of primary iron overload (Powell et al., 2005), while secondary iron overload is usually caused by the introduction of excess iron in the body. Excess iron can result in iron overload anemia (thalassemia, congenital anemia, and myelodysplastic syndrome, etc.), and blood transfusion may be needed under these conditions (Heimpel et al., 2003; Meerpohl et al., 2014; Taher and Saliba, 2017; Hoeks et al., 2018). Excessive circulating iron exceeds the buffering capacity of transferrin (Tf). This results in cellular and tissue oxidative damage and organ dysfunction (Abraham and Kappas, 2005). It was found that the level of iron ions was significantly high in the synovial fluid (Yazar et al., 2005), and there was an accumulation of hemosiderin on the synovium of OA patients (Ogilvie-Harris and Fornaiser, 1980). It was also observed that the level of serum ferritin in patients suffering from OA could be positively correlated with the degree of damage of knee cartilage (Kennish et al., 2014). Iron overload represents the situation where an excess of iron accumulates in the body. This can potentially cause damage to cells. Cell damage can be attributed to peroxide stress, and pathological changes of various tissues may occur under these conditions (Brissot et al., 2019). Therefore, it is important to understand the role of iron in the development of OA under conditions of iron overload.

**FIGURE 1:**
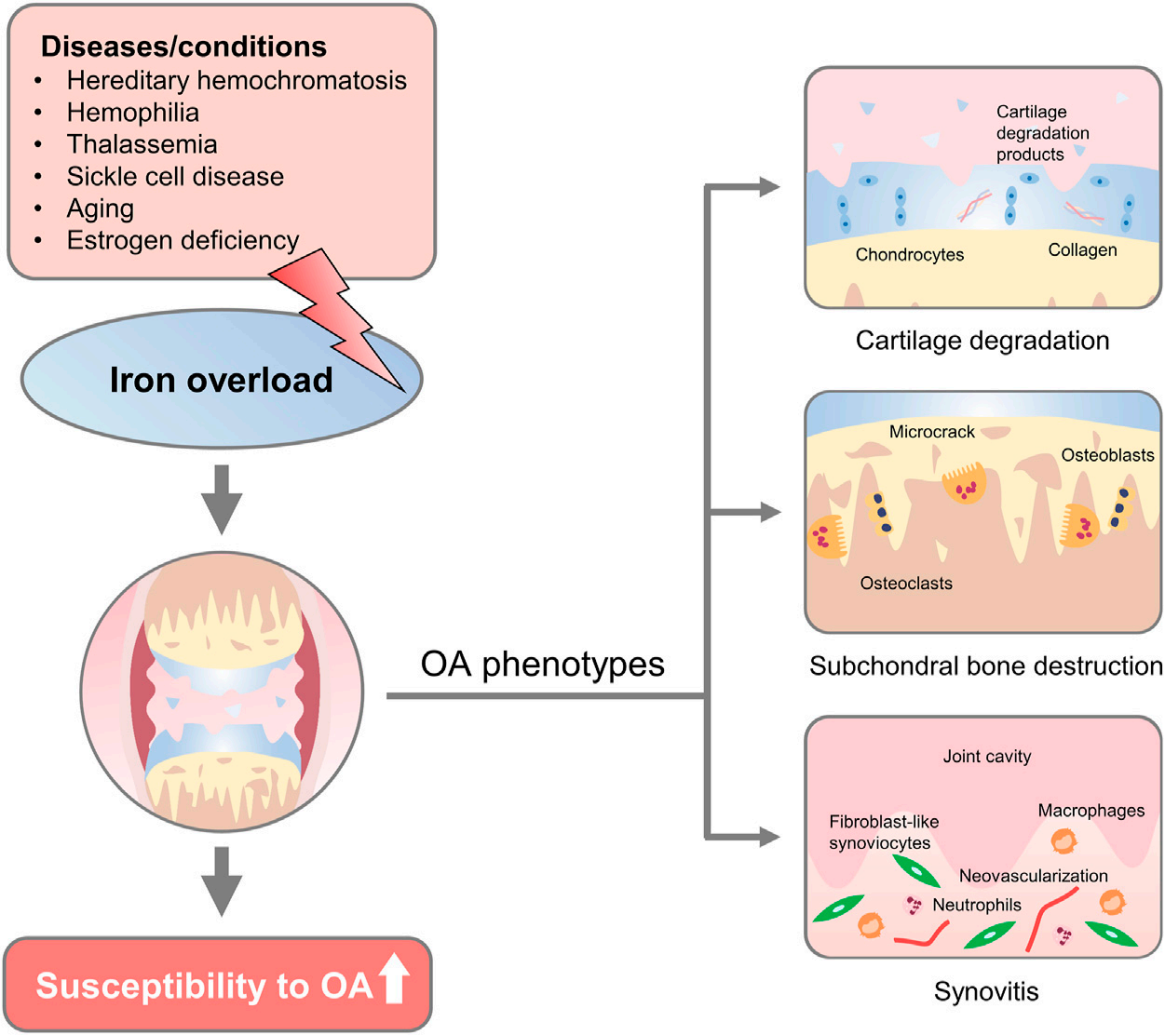
Overview of iron overload etiologies and osteoarthritic phenotypes. Diseases/conditions with diverse etiology, such as hereditary hemochromatosis, hemophilia, thalassemia, sickle cell disease, aging, and estrogen deficiency could lead to iron overload in the human body. Changes that affect iron overload in the joint increase the susceptibility to developing osteoarthritic phenotypes, including progressive cartilage degradation, altered microarchitecture and biomechanics of subchondral bone, and persistent joint inflammation, proliferative synovitis, and synovial pannus.

This review presents the relationship between iron overload and OA progression. Herein, the progress made in the field has also been presented. We have also discussed the clinical significance and cellular mechanisms of iron overload-associated OA to determine novel prophylactics, therapies, and predictive values that can be used for the treatment of OA **(** Figure 1 **)**.

## 2 Detrimental Role of Iron Overload on Joint Homeostasis and Function

Clinical observations revealed that osteoarthritic phenotypes such as progressive cartilage degradation, altered microarchitecture and biomechanics of subchondral bone, persistent joint inflammation, proliferative synovitis, and synovial pannus are the common characteristics associated with iron overload (Nieuwenhuizen et al., 2013; Tchetina et al., 2016; Mobasheri et al., 2017). The osteoarthritic phenotypes have been found in multiple animal models used to study iron overload (Carroll, 2006; Burton et al., 2020). This indicated the adverse effects of excess iron on joint homeostasis and joint function. Much work has been done to understand the effect of iron overload on the progression of OA (Simão et al., 2019; Jing et al., 2020), but the specific relationship between iron overload and progression of OA is yet to be completely understood. The clinical significance of iron overload and the cellular mechanisms involved with OA associated with iron overload should also be researched further. In this section, we have discussed the observations made during the study of iron overload-associated OA. The results obtained by conducting clinical research and studying animal models have been presented.

### 2.1 Clinical Findings

#### 2.1.1 Osteoarthritic Joint Complications in Hereditary Hemochromatosis

The term hemochromatosis encompasses a group of disorders caused by iron overload. HH is the most common disorder belonging to this group that is caused by the mutation of the homeostatic iron regulator (HFE) gene (Alqanatish et al., 2021). Most patients suffering from HH in northern Europe are homozygous. In these cases, missense mutations can be observed at position 282 of the HFE protein (C282Y) (McLaren and Gordeuk, 2009). Another type of HFE gene mutation results in a change in the 63 amino acids (H63D) (Milman et al., 2004; Gunel-Ozcan et al., 2009). It has also been observed that other subtypes of HH can be caused by mutations in the hepcidin antimicrobial peptide (HAMP) gene-encoding hepcidin or the main inducer genes (such as HFE, transferrin receptor-2 gene (TfR2), and hemojuvelin gene (HJV)) associated with hepcidin expression (Camaschella et al., 2000; Roetto et al., 2003; Papanikolaou et al., 2004).

HFE gene mutations are primarily characterized by systemic iron overload in multiple tissues, such as the tissues in the liver, heart, and kidney (Wagner et al., 2019). It has been recently reported that the iron content increased consistently in patients suffering from HH (Kent et al., 2015), However, the researchers did not explore the relationship between iron levels and the severity of the arthritic disease. Ferritin is currently considered the “gold standard” for iron contents (Abtahi et al., 2019). Ferritin levels in the synovial fluid under conditions of HFE gene mutation in patients suffering from OA were found to be higher than the ferritin levels recorded in HFE wild-type OA patients (Carroll et al., 2010). Additionally, high levels of serum ferritin indicated that HH was characterized by a clinically definable arthropathy that could be attributed to iron overload. It was also revealed that iron overload was likely to be a critical determinant of joint disease in patients suffering from HH (homozygosity for C282Y) (Carroll et al., 2011). Recently, results obtained from a case-control study revealed that patients with HH were at a higher risk of suffering from OA than the members belonging to the control groups (50.5 vs 28.9%). Specifically, relative to controls, HH patients were at a higher risk of needing knee and hip replacement prostheses. The degree of iron overload (ferritin concentration >1000 μg/L) influenced OA progression. This suggested that severe joint complications could be observed in patients with substantially elevated ferritin levels (Richette et al., 2010).

#### 2.1.2 Arthritic Manifestations of Thalassemia

Iron overload-linked anemia is characterized by ineffective erythropoiesis that causes hepcidin inhibition. This, in turn, results in a high degree of absorption of dietary iron and secondary iron overload (Gupta et al., 2018). A typical case is β-thalassemia caused by the abnormal β-globin formation and the subsequent apoptosis of mature erythrocytes (Manolova et al., 2019). Consequently, this stimulates the production of erythropoietin in the human body, and an increase in the levels of immature erythroid precursors can also be observed. It has also been observed that erythropoiesis is ineffective, and the degree of continues to increase (Ribeil et al., 2013). An increase in the number of erythroid precursors results in an increase in the secretion of erythroid regulatory factors. This results in the inhibition of hepcidin and an increase in the iron levels that promotes the production of erythrocytes, resulting in increased levels of iron absorption (Kautz et al., 2015; Liu et al., 2019). In addition, regular blood transfusion therapy is required for thalassemia major patients to maintain adequate hemoglobin concentrations (Viprakasit and Ekwattanakit, 2018). The ability of the human body to actively excrete excess iron is limited. Hence, long-term blood transfusion could result in iron overload, despite the use of iron chelators during the process of blood transfusion (Shah et al., 2010; Al-Hakeim et al., 2020). This results in excess iron deposits in various organs (especially in the pancreas, liver, and heart) and sometimes in the joints (Altinoz et al., 2012).

Thalassemia-associated arthritic manifestations are one of the most common co-morbidities in thalassemia patients (Noureldine et al., 2018). It has also been observed that iron overload is one of the primary manifestations of thalassemia, which to a large extent leads to the occurrence of terminal organ joint complications in thalassemia patients. A study conducted as early as the 1970s involved the examination of arthropathy in 50 transfusion-dependent β-thalassemia patients of European descent (Gratwick et al., 1978). Among these participates, 18 patients developed mild joint pain (after exercise) that lasted several days. Four patients suffered from severe symptoms lasting for at least 3 months, and they were not capable of walking to school or work frequently. Pain in the joints of calves, ankles, or forefeet was reported. Bone histomorphometry revealed microfractures, osteomalacia, and increased intercellular spaces between osteoblasts and osteoclasts in lesions of osteoarthropathy. Interestingly, iron deposits were detected along the calcification front and cement lines, with possible contributions from iron overload to the pathophysiology of the joint disease. In the late 20th century, prior to the invention of iron-chelating drugs, ankle OA in patients with thalassemia was frequently reported. Bone marrow dilatation, iron deposition in the joint microenvironment, or hypoparathyroidism, which together led to OA, were often observed in these patients (Noureldine et al., 2018; Dhawan et al., 2020; Quarta et al., 2020).

#### 2.1.3 Sickle Cell Disease- and Hemophilia- Associated Osteoarthritis

SCD is one of the most common autosomal recessive blood hereditary diseases, affecting approximately 300,000 newborns every year (Abraham et al., 2016). Single nucleotide mutation in the Hb β-chain coding gene is responsible for the molecular mechanism of this disease occurrence (Dever et al., 2016; Kato et al., 2018). SCD-associated OA is generally multiarticular and symmetrical and tends to occur in large joints and lower extremities. Radiographs reveal reduced periarticular bone mass, joint space narrowing, and synovial inflammation (Vanderhave et al., 2018). Clinical findings have revealed that the iron levels of SCD patients could not be accurately determined. Some researchers have linked SCD to iron overload, while others have observed iron deficiency in SCD patients (Koduri, 2003; Koren et al., 2010). Interestingly, this phenomenon could be partly explained by the fact that the level of iron deposition varied significantly from patient to patient. The variations could be observed by conducting an autopsy (Natta et al., 1985). Nevertheless, results from recent clinical retrospective research works revealed that approximately 70% of patients suffering from SCD and exhibiting high serum iron levels had a relatively low bone mass, indicating that iron overload (observed under these conditions) may have an adverse effect on osteochondral homeostasis (Sadat-Ali et al., 2011).

Although clinical findings have revealed that osteoarthritic phenotypes are one of the common complications in patients with hemophilia (Braner, 2018), related pathogenesis and mechanisms of disease progression are yet to be fully understood. Despite this, the frequent occurrence of OA in patients suffering from hemophilia has led to a growing curiosity about the role of iron in promoting OA. Repeated bleeding from the same joint leads to progressive joint damage and the development of hemophilic arthropathy. This results in joint pain, deformity, and disability (Yoo et al., 2009). Iron overload-mediated pathogenesis of hemophilic arthropathy is associated with multifactorial pathological processes. Hemophiliac arthropathy is a type of secondary OA. The deposition of iron in the joints can directly lead to the degeneration of synovium, cartilage, and subchondral components. Von Drygalski et al. established the relationship between cartilage hemosiderin in hemophilic joints and joint deterioration using the innovative joint-specific MRI T2* sequences (von Drygalski et al., 2019). Moreover, besides the accumulation of iron in joints (a critical factor that influences the process of cartilage degradation), bone structure bleeding, hemosiderin deposition, and angiogenesis in subchondral cysts were also observed in 78% of the joints of the patients suffering from hemophilia (Zhou J. Y. et al., 2021).

#### 2.1.4 Aging- and Estrogen Deficiency- Induced Osteoarthritis

Although age, mechanical load, and joint injury are the primary risk factors, pathophysiology and internal mechanisms associated with OA have not been fully explored (Shane Anderson and Loeser, 2010; Courties et al., 2015). During the aging process, iron accumulates in various tissues and organs due to the lack of the main mechanism of iron excretion in the human body (Mangan, 2021). Thus, it is speculated that the degenerative cartilage changes in middle-aged and elderly patients with OA may be related to iron overload in the joints. It is believed that the level of iron significantly influences age-related diseases because iron can promote the generation of free radicals. Multiple independent clinical studies have been conducted, and the results have indicated that elderly patients with high ferritin content have a four-fold increased risk of suffering from OA than patients with low ferritin content in their bodies. It has also been observed that the ferritin content has a positive association with the severity of joint through imaging analyses (Park et al., 2012; Jing et al., 2021a; Ke et al., 2021). Moreover, studies indicated that the incidence of high iron storage was higher in the elderly compared with populations of other ages, and this transition was more pronounced in older women than in men (Chen et al., 2015). This phenomenon may be related to estrogen deficiency in aged women under post-menopausal conditions (Ko and Kim, 2020).

Estrogen deficiency, observed during menopause, is currently considered a critical cause of menopausal manifestations and symptoms (Kim and Kim, 2019). During menopause, significant changes in metabolism occur in the human body. In addition to multiple endocrine and hormonal changes, changes in the process of iron metabolism are also observed during the complex and delicate process of the menopausal transition (Trenti et al., 2018; Shin et al., 2020). The serum ferritin concentrations in postmenopausal women were two-to three-fold higher than the ferritin concentrations in women not in their menopausal stage. The levels are roughly similar to the reduced levels of estrogen (Park et al., 2012; Ke et al., 2021). It has been widely reported that besides a significant reduction in estrogen production, an excessive increase in iron or ferritin levels can also pose health risks in postmenopausal women (Liu et al., 2006; Kim et al., 2012; Feldbrin et al., 2016). Thus, the relatively higher incidence and susceptibility of old women toward OA compared to men could be explained, and this could also be attributed to iron overload (Zhang et al., 2001). To some extent, the cessation in reproductive function also decreases iron loss associated with menstruation or pregnancy (Breymann, 2015; Pedlar et al., 2018). The researchers presented clinical evidence that increased systemic iron can potentially be an independent adverse factor in the progression of OA in postmenopausal women.

#### 2.1.5 Comparison in Term of Iron Metabolism and Osteoarthritis Severity

Iron metabolism and homeostasis dysregulation are commonly observed in multiple inherited blood diseases. These are also observed in the elderly and patients suffering from estrogen deficiency. However, there may exist more commonalities or non-commonalities in the changes in iron metabolism and its subsequent influence and the manifestation or progression of OA caused by these different diseases. In terms of iron metabolism regulation, HH, aging, and estrogen deficiency are characterized by abnormal processes of iron acquisition and efflux. This dysregulation is largely attributed to the involvement of the hepcidin-ferroportin (FPN) axis. HH results in inappropriately low hepcidin levels and unregulated FPN activity. Under these conditions, the inability to inhibit the absorption of iron from the diet and the release of iron from macrophages are also observed. This results in the accumulation of iron in various tissues (Papanikolaou et al., 2004). In patients suffering from thalassemia, hepcidin is inhibited to increase the availability of iron due to ineffective erythropoiesis, resulting in excessive iron absorption and iron overload (Camaschella and Nai, 2016). It has also been reported that estrogen regulates iron homeostasis by regulating the expression of hepatic hepcidin via an estrogen response element (Hou Y. et al., 2012). Iron overload observed under conditions of hemophilia is responsible for erythrocyte lysis-derived iron. This results in uncontrolled intra-articular capillary bleeding in hemophilic patients post exercise (Cooke et al., 2018). Blood transfusion (also the reason for thalassemia-related iron overload) plays a prominent role in the management of patients with SCD, but causes significant iron overload (Porter, 2009). It has been inferred that changes in the pathobiological iron overload are triggered by multiple diseases or conditions. However, the progression and severity of joint deterioration have not been studied in detail, and these fields should be explored further.

### 2.2 Animal Models

Osteoarthritic phenotypes have been noticed in multiple animal models subjected to conditions of iron overload. This indicated the adverse effect of iron overload on the development and progression of OA. Firstly, osteoarthritic phenotypes were studied in some genetic iron overload animal models. Camacho et al. used Hfe knockout (KO) mice suffering from HH and explored the progression of OA in mice under conditions of iron overload. The results suggested that iron overload accelerated the progression of OA (Camacho et al., 2016). In another study, researchers investigated the effect of high iron content on the articular chondrocytes isolated from Hfe KO mice and compared the results with the results obtained by studying wild-type mice (Simão et al., 2019). The results indicated that primary chondrocytes from both sources, when exposed to excessive exogenous iron, exhibited a cellular phenotype similar to OA. Increased metalloproteinase contents and decreased extracellular matrix protein levels were observed. Hfe-KO chondrocytes revealed a favored expression of iron metabolism markers (such as Tf), indicating increased sensitivity to intracellular iron contents (Simão et al., 2019). In summary, the presence of excessive iron affects the process of chondrocyte homeostasis under conditions of Hfe -KO. These samples are more likely to exhibit a cell phenotype similar to that observed in samples subjected to conditions of OA.

To elucidate the direct effect of iron overload on osteoarthritic phenotypes, iron-overload mouse models were developed by injecting iron dextran. Lindsey et al. established a close relationship between iron overload and OA by injecting iron dextran into guinea pigs characterized by a low incidence of OA (Burton et al., 2020). Excessive iron exposure exacerbated the severity of knee OA, and this was validated by the results obtained by analyzing micro-CT and conducting histological assessments. The association between iron overload and OA was demonstrated by the study conducted by Jing et al., who used iron overloaded mice for their studies. Severe cartilage destruction, increased expression of matrix metalloproteinase-13 (MMP13), disintegrin and metalloproteinase with thrombospondin motifs 5 (ADAMTS5), and increased iron levels in the circulating blood were observed in iron-overloaded mice (Jing et al., 2020).

## 3 Cellular and Molecular Mechanisms of Iron Overload Involved in Osteoarthritis

**FIGURE 2:**
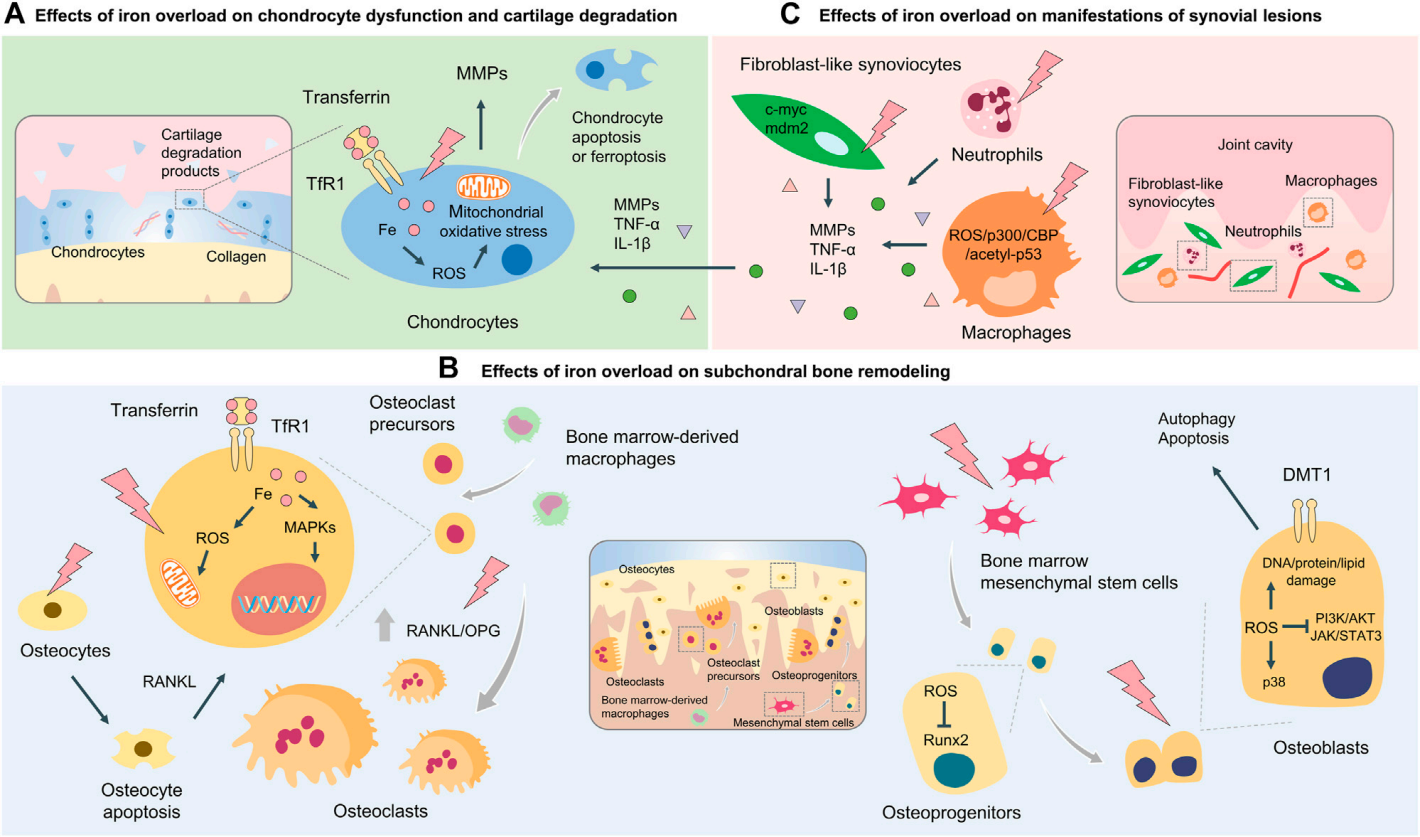
Cellular and molecular mechanisms of iron overload involved in OA. Cellular and molecular mechanisms of iron overload involved in OA include the effects of iron overload on chondrocyte dysfunction and cartilage degradation **(A)** subchondral bone remodeling process which osteoclasts and osteoblasts coordinately regulate **(B)**, and manifestations of synovial lesions mediated by macrophages, fibroblast-like synoviocytes, and neutrophils **(C)**.

In the previous chapter, we focused on the findings of iron overload-related OA in animal models, and the results suggested that iron overload was closely related to the occurrence and progression of OA. The question to be asked is, “At the cellular level, which cell types are affected by iron overload that promotes OA progression?” Research works in the field of the pathophysiology of OA have focused on articular cartilage. It is being increasingly believed that OA affects the whole joint tissues, including the subchondral bone and synovial lining of the articular cavity (Egloff et al., 2012). During the occurrence of OA, cartilage and subchondral bone damage results in the disruption of the anabolic/catabolic metabolism of chondrocytes. The imbalance between osteoclasts and osteoblasts involved in abnormal subchondral bone remodeling is also observed in these conditions (Yu et al., 2016). Inflammatory “synovitis” in OA encompasses various abnormalities, such as synovial lining hyperplasia, infiltration of macrophages, pannus formation, and fibrosis (Wei and Bai, 2016). Under conditions of iron overload, the effects of iron on chondrocytes, osteoclasts, osteoblasts, and the cells in the synovial lining have been recently investigated *in vitro*. The results will be discussed in this chapter (Figure 2; Table 1).

**TABLE 1:** Cellular and molecular mechanisms underlying the interplay between iron overload and OA.

Osteoarthritic phenotypes	Cells	Mechanisms	References
Cartilage degradation	Chondrocytes	Promote lipid peroxidation and stimulate ferroptosis	Sun et al. (2020)
		Mediate mitochondrial dysfunction through ROS production and oxidative stress response	Jing et al. (2021a)
Subchondral bone destruction	Osteoclasts	Promote mitochondrial respiration and oxidative stress, and thus facilitate osteoclast differentiation	Ishii et al. (2009)
		Facilitate osteoclastogenesis by promoting the secretion of apoptotic osteocyte-derived RANKL	Yang et al. (2020)
	Osteoblasts	Inhibit osteogenic differentiation of BMSCs through ROS-mediated Runx2 suppression	Jing et al. (2019)
		Induce osteoblast autophagy and apoptosis by upregulating the level of DMT1	Liu et al. (2017)
		Promote osteoblast apoptosis by inducing ROS production and oxidative stress injury	He et al. (2013)
		Lead to G1 phase arrest and autophagy by inhibiting PI3K/AKT and JAK/STAT3 signaling and promoting p38 signaling	Cen et al. (2018)
Synovitis	Macrophages	Induce M1 polarization by increasing ROS-stimulated p300/CBP acetyltransferase activity and p53 acetylation	Zhou et al. (2018)
	Neutrophils	Promote the accumulation of neutrophils and regulate matrix-degrading enzymes production	Heiland et al. (2010)
	Fibroblast-like synoviocytes	Promote cell proliferation by activating key genes c-myc and mdm2	Hakobyan et al. (2004)

### 3.1 Effects of Iron Overload on Chondrocyte Dysfunction and Cartilage Degradation

In articular cartilage, chondrocytes are the sole cell types that participate in the processes of synthesis and renewal of the extracellular matrix. They also help in the maintenance of matrix integrity (Musumeci et al., 2015; Yao et al., 2021). Impairment and function of chondrocytes result in progressive damage of the articular cartilage (Hwang and Kim, 2015; Musumeci et al., 2015). Chondrocyte apoptosis is one of the pathological factors that cause degenerative changes in articular cartilage. It has also been observed that the imbalance of cartilage metabolism in chondrocytes results in the secretion of matrix-degrading enzymes such as MMPs and ADAMTS, thus compromising the integrity of the cartilage matrix (Verma and Dalal, 2011; Mehana et al., 2019). MMP-13 is one of the most important enzymes in MMPs targeting cartilage degradation. Clinical studies have shown that the chondrocytes in OA patients with articular cartilage injury greatly express MMP-13 (Salerno et al., 2020). A variety of cytokines can also affect the progression of OA by affecting chondrocyte metabolism. Inflammatory cytokines, such as tumor necrosis factor-α (TNF-α) and interleukin-1β (IL-1β), also play a vital role in the development of OA (Wojdasiewicz et al., 2014; Wang and He, 2018). IL-1β is one of the most important pro-inflammatory factors, which can significantly upregulate the expressions of MMPs and accelerate cartilage degeneration under conditions of OA (Jenei-Lanzl et al., 2019).

A large number of observations suggest that an excess of iron facilitates chondrocyte apoptosis and promotes the upregulation of the expression of matrix-degrading enzymes. Jing et al. used ferric ammonium citrate (FAC) to establish an excess iron condition, and they reported that the presence of excess iron accelerated chondrocyte apoptosis, resulting in the expression of matrix-degrading enzymes such as MMP-3 and MMP-13 (Jing et al., 2020). Results have also revealed that chondrocytes from Hfe-KO mice were more likely to develop OA-related phenotypic characteristics, such as upregulation of MMPs expression, decreased production of extracellular matrix, and decreased expression of aggrecan. Hfe-KO chondrocytes promoted the upregulation of key proteins associated with iron metabolism (such as Tf), suggesting an increased sensitivity to the process of iron deposition (Simão et al., 2019). Furthermore, an excess of iron affected chondrocyte homeostasis in models subjected to Hfe gene KO which were more likely to exhibit a cell phenotype similar to that observed under conditions of OA (Simão et al., 2019). Iron overload facilitated the oxidative stress response of chondrocytes. Moreover, when the amount of intracellular iron was in excess, hydroxyl free radicals were produced under conditions of the Fenton reaction. Under the reaction conditions, lipid peroxidation was promoted, and ferroptosis, a new form of programmed cell death, was induced (Sun et al., 2020). Ferroptosis is a process of cell death that occurs when an excess of iron and reactive oxygen species (ROS) accumulate. The process is triggered by the inactivation of the cellular glutathione-dependent antioxidant defense system (Hirschhorn and Stockwell, 2019). Yao et al. reported that chondrocytes readily underwent ferroptosis in the environment of inflammation and excess iron (Yao et al., 2021). They used IL-1β and FAC to establish the inflammatory environment and generate conditions of iron overload for chondrocytes, respectively. Increased levels of lipid ROS and upregulated ferroptosis-related protein expression, which further led to an increase in the type collagen II expression and a decrease in the MMP-13 expression, were also observed under these conditions. Furthermore, surgically treated OA mice exhibited a reduced protein expression of GPX4 in articular cartilage. Additionally, intra-articular administration of ferrostatin-1 (a ferroptosis-specific inhibitor) in mice induced with OA rescued the protein expression of GPX4 and type collagen II. This resulted in improved cartilage erosion (Yao et al., 2021). However, the molecular mechanisms of ferroptosis in chondrocytes are not well defined. Recently, HIF-2α was reported to augment lipid storage and lipid peroxide accumulation. It was also reported that HIF-2α promoted CPT1A-mediated β-oxidation, and therefore it was identified as the central mediator of the ferroptosis of chondrocytes. Furthermore, overexpression of HIF-2α in chondrocytes attenuated the expression of GPX4 and exacerbated cartilage degeneration. These findings implied that ferroptosis could be potentially associated with the progression of OA (Zhou X. et al., 2021).

Recently, the role of oxidative stress and mitochondrial dysfunction in OA development has gradually attracted the attention of researchers (Wang F.-S. et al., 2020). However, the effect of mitochondrial dysfunction on chondrocyte metabolism under conditions of excess iron remains to be explored. Another study by Jing et al. revealed that excess iron-mediated mitochondrial dysfunction in chondrocytes had a close association with oxidative stress. The association was established via the process of ROS generation, which in turn promoted the expression of OA-related catabolic markers (Jing et al., 2021a). The iron chelation method further confirmed the vital role of iron in chondrocyte dysfunction. Calcium chelator, which can repress the influx of iron by regulating the extent of internalization realized for the transferrin receptor (TfR), is emerging as an effective drug for treating iron overload-related diseases. Based on the theory that iron overload promotes OA by inducing ROS production and mitochondrial dysfunction, Han et al. further revealed that the calcium chelator, BAPTA-AM, suppressed the influx of iron into chondrocytes and effectively restrained iron overload-mediated ROS accumulation and mitochondrial dysfunction. This suggested that calcium chelators played important roles during the treatment of iron metabolism-related OA (Jing et al., 2021b).

### 3.2 Effects of Iron Overload on Subchondral Bone Remodeling

Although cartilage loss is considered the primary cause of OA, an increasing number of research works have revealed that the integrity of the subchondral bone structure and subchondral bone homeostasis play indispensable roles in the occurrence and development of OA (Hu et al., 2020). Under normal conditions, subchondral bone undergoes a dynamic remodeling process in which osteoclasts and osteoblasts jointly regulate and maintain the process equilibrium (Tian et al., 2021). Under conditions of abnormal mechanical stress, the extent of differentiation and activation of subchondral bone osteoclast precursors was realized, and the subchondral bone resorption activities significantly increased (Fang et al., 2021). Under these conditions, the subchondral bone shows osteoporotic changes (such as decreased bone mass, increased trabecular space, and thinning of the subchondral bone plate). These changes put a greater stress load on the upper hyaline articular cartilage, resulting in the progression of OA.

Osteoclasts are multinucleated giant cells differentiated from mononuclear macrophages. They participate in the process of bone absorption (Kylmaoja et al., 2016). Numerous researchers have reported that abnormal iron metabolism is closely related to osteoclast formation and differentiation. Jia et al. reported that FAC facilitated receptor activator of nuclear factor-kappa B ligand (RANKL)-mediated osteoclastogenesis in RAW264.7 cells and bone marrow-derived macrophages (BMMs). Oxidative stress, resulting from excessive ROS production, hindered the process of homeostasis by stimulating osteoclastogenesis and inhibiting the functions of osteoblasts (Jia et al., 2012). It has been recently reported that iron overload can potentially cause an increase in the extent of mitochondrial respiration and induce an oxidative stress response in osteoclasts (Ishii et al., 2009). The process of iron chelation was conducted, and the results further confirmed the critical role of iron in the process of osteoclast maturation. Results from a recent *in vitro* study illustrated that lactoferrin, an iron-binding glycoprotein, effectively suppressed the process of osteoclast differentiation of monocytes and significantly increased the extent of bone formation in adult mice (Cornish et al., 2004). The RANKL to osteoprotegerin (OPG) ratio is a critical factor determining osteoclast differentiation. In addition, iron overload conditions usually resulted in an elevated RANKL/OPG ratio. Hou et al. reported that the iron-chelating lactoferrin could improve bone density by increasing the extent of OPG generation (Hou J.-m. et al., 2012). Iron uptake of Tf-bound Fe3+ is facilitated via TfR1. Ishii et al. reported the relationship between the expression of TfR1 and osteoclast differentiation. They reported that TfR1-mediated iron influx promoted osteoclastogenesis and bone-resorbing capacity, and iron chelator deferoxamine (DFO) hindered this process in a dose-dependent fashion (Ishii et al., 2009). Further research indicated that DFO directly suppressed iron overload-induced osteoclastogenesis by negatively regulating the mitogen-activated protein kinase (MAPK) signaling pathway, which was independent of the process of ROS activation (Zhang et al., 2019). Interestingly, osteocytes were also found to participate in the process of bone homeostasis by regulating the activities of osteoclasts and osteoblasts in the bone microenvironment. Yang et al. were the first to report that an excess of iron indirectly regulated the process of osteoclast differentiation by regulating osteocyte activity. The results reported by Yang et al. indicated that the process of osteocyte apoptosis induced by iron overload effectively facilitated osteoclastogenesis by promoting the secretion of osteocyte-derived RANKL (Yang et al., 2020).

Bone marrow mesenchymal stem cells (BMSCs) are adult stem cells present in the bone marrow cavity. They are capable of self-renewal and multidirectional differentiation and significantly affect human development, the onset of aging, and the occurrence of diseases (Baker et al., 2015). Osteogenic differentiation of multipotent BMSCs also plays a crucial role in the process of subchondral bone remodeling (Wang J. et al., 2020). Iron negatively influences the process of bone homeostasis and disrupts the proliferation capacity and differentiation balance of BMSCs. Results from current studies suggest that an excess of iron hinders the process of osteoblast differentiation and activity, and impaired extracellular matrix mineralization is realized under these conditions. Balogh et al. studied the effect of iron and ferritin on the process of osteoblast differentiation of BMSCs. The results revealed that iron and exogenous ferritin significantly inhibited the process of osteoblast differentiation of BMSCs (Balogh et al., 2016). The relationship between increased ferritin and downregulated expression of runt-related transcription factor 2 (Runx2) in compact bone osteoprogenitor cells was studied in mice under conditions of iron overload (Balogh et al., 2016). Liu et al. reported that excess iron-induced osteoblast autophagy and apoptosis by upregulating the levels of divalent metal transporter 1 (DMT1), an iron transporter (Liu et al., 2017). It was also reported that an excess of iron interfered with the normal metabolism of osteoblasts by inducing oxidative stress response under conditions of increased intracellular iron levels. An excess of ROS was generated under conditions of iron overload (following Fenton reaction). The balance of cellular antioxidant and oxidant levels was lost, and this damaged DNA, protein, and lipid structures inducing osteoblast apoptosis (He et al., 2013). In addition, existing results have shown that iron overload results in the generation of ROS. The phosphatidylinositol 3-kinase (PI3K)/AKT and Janus kinase (JAK)/signal transducer and activator of transcription 3 (STAT3) signaling pathways were hindered, and the p38-MAPK pathway was promoted under these conditions, resulting in G1 phase arrest and autophagy in the osteoblast cell line MC3T3-E1 (Cen et al., 2018). The above findings indicated that excessive bone resorption and abnormal bone formation might be involved in the underlying mechanisms associated with iron overload-related subchondral bone instability.

### 3.3 Effects of Iron Overload on Manifestations of Synovial Lesions Mediated by Various Cell Types

OA is a degenerative inflammatory disease, leading to multiple joint and peri-joint damage (Robinson et al., 2016). Inflammatory “synovitis” in OA (a form of periarticular tissue damage) encompasses various abnormalities, such as synovial lining hyperplasia, infiltration of macrophages, pannus formation, and fibrosis (Sellam and Berenbaum, 2010). These manifestations of synovial lesions are mediated by various cell types in the synovial lining of the joint cavity (Scanzello and Goldring, 2012). In iron overload conditions, cells (such as macrophages, fibroblast-like synoviocytes (FLSs), and neutrophils) present in the synovial lining exhibit pathological features.

An *in vivo* study of hemophilia indicated that iron-overloaded synovial tissue released pro-inflammatory cytokines (such as IL-1β and TNF-α) that stimulated catabolic activities in chondrocytes and accelerated the degenerative pathological changes in cartilages associated with OA (Nieuwenhuizen et al., 2013). The inflammatory response produced by articular and periarticular tissue, one of the major factors accelerating the process of OA progression, is predominantly the skew of the macrophage polarization towards the M1 phenotype (Mahon et al., 2020). Macrophages are derived from monocytes and are widely present in the synovial tissues of joints (Zhang et al., 2020). Polarized M1 macrophages could induce synovial inflammation, affecting chondrocyte metabolism and resulting in the degradation of the cartilage matrix (Wu et al., 2020). Zhou et al. reported that the process of iron-mediated ROS production promoted the process of M1 macrophage polarization by increasing the p300/CBP acetyltransferase activity and facilitating the process of p53 acetylation (Zhou et al., 2018).

Chen et al. qualitatively compared rheumatoid arthritis (RA), OA, and HH tissues and found that neutrophil invasion was significantly high under conditions of HH-related arthropathy. This was pronounced in joints containing high levels of iron deposits. These results suggested that the accumulation of neutrophils could potentially regulate the production of stromal enzymes, resulting in cartilage degradation and rapid progression of joint injury (Heiland et al., 2010). Moreover, iron participates in the initiation of the growth of synovial pannus (Mendonça et al., 2016). OA is a degenerative inflammatory disease characterized by pannus tissues consisting of FLSs, macrophages, and lymphocytes (Duc et al., 2008). Among these cells, FLSs are special types of cells present in hyperplastic synovial pannus tissues. These cells cause articular damage by secreting cytokines, chemokines, and matrix-degrading proteins (Chang et al., 2010; Raychaudhuri et al., 2018). However, little information about iron overload in FLSs is available. Hakobyan et al. reported that iron promoted the process of cell proliferation in human and mouse synovial tissues, and the expression of key genes such as c-myc and mdm2 were responsible for the proliferation of synovial cells. Under these conditions, the occurrence and development of vascular synovitis were also promoted (Hakobyan et al., 2004).

## 4 Potential Clinical Interventions and Value of Iron Overload in Osteoarthritis

### 4.1 Predictive Biomarker

As reported in the literature, diverse pathologies or conditions, such as HH, hemophilia, thalassemia, SCD, aging, and estrogen deficiency, could result in iron overload in the human body. Changes that result in iron overload in the joints increase the chances of developing osteoarthritic phenotypes. These results revealed the significance of iron overload on the process of OA onset. Richette et al. observed that patients suffering from HH had a higher chance of suffering from OA than patients belonging to the control group (Richette et al., 2010). Patients with HH had a higher risk of needing knee and hip replacement compared to patients belonging to the control groups. The results from the case-control study strongly suggested that joint damage could be more severe in patients with increased ferritin levels (Richette et al., 2010). Results from clinical studies also revealed that the risk of suffering from OA increased 4-fold in the elderly with high ferritin contents in the serum compared to patients belonging to other age groups. The results from imaging experiments revealed that the level of serum ferritin correlated positively with the severity of joint damage (Park et al., 2012; Ke et al., 2021). Thus, further studies on serum iron parameters that can be used for predicting OA should be conducted. The results can potentially help in predicting the progression of OA.

### 4.2 Prophylactic Intervention

As iron overload accelerates the progression of OA under various conditions, early reduction of iron intake (such as diet adjustment) or the daily temperate promotion of iron excretion can potentially be a prophylactic intervention. Under conditions of aging, HH, hemophilia, etc., iron promotes the progression of OA as the presence of iron triggers the production of free radicals (Emerit et al., 2001; Jomova and Valko, 2011). It has been observed that serum ferritin concentrations increase by two-to three-fold in postmenopausal women. This reflects a drop in the estrogen levels (Milman and Kirchhoff, 1992; Zacharski et al., 2000). In postmenopausal women, increased total iron contents in the body could accelerate the development of OA. Thus, the elderly, as well as the patients exhibiting mild OA symptoms, or people with a family history of HH or hemophilia could be studied for monitoring the serum iron parameters and realizing prophylactic intervention. However, the process is a potential double-edged sword. Iron is an indispensable nutritional element present in the body and a part of many important macromolecules involved in energy production, respiration, DNA synthesis, and metabolism (Anderson and Frazer, 2017; Gao et al., 2019). Maintaining proper “free iron” levels is a key part of achieving a balanced iron metabolism (Gulec et al., 2014; Muhoberac and Vidal, 2019). Due to this dual nature of iron, serum iron contents need to be monitored regularly for ensuring normal iron levels in the body while realizing preventive intervention methods to address the problem of iron overload.

### 4.3 Therapeutic Strategy

Iron chelation therapy can potentially be an effective method of treating patients with systemic iron overload (Gordeuk et al., 1994). The effectiveness of iron chelators (such as DFO, deferiprone, and deferasirox) has been widely validated in iron overload diseases in patients requiring long-term transfusions and suffering from HH, SCD, and thalassemia (Borgna-Pignatti et al., 1998; Lal et al., 2013; Ballas et al., 2018). Although the pathogenesis of OA in patients with iron overload has not been fully explained, the adverse effects of iron overload on cells present in the joint and the progression of OA has been extensively reported (Depierreux et al., 1988; Barg et al., 2011; Jing et al., 2021a). Therefore, the process of iron chelation therapy can be potentially used to shed light on the process of the prevention or treatment of iron overload-related OA. In addition, we have found evidence by conducting cellular-level studies and using animal models that the process of iron chelation can be used to effectively maintain joint homeostasis. The process can also help alleviate the progression of OA. Oxidative stress and mitochondrial dysfunction induced by iron overload result in chondrocyte apoptosis and metalloproteinase upregulation. The iron chelator DFO could significantly inhibit these processes (Jing et al., 2021a). An *in vitro* study was conducted recently, and the results revealed that the protein levels of MMP-3 and -13 in primary chondrocytes increased significantly following IL-1β treatment. Pretreatment with iron-chelating DFO effectively reversed the process of upregulation (Jing et al., 2020). These results suggest that the process of iron chelation can potentially help in preventing the apoptosis of chondrocytes and the destruction of the cartilage extracellular matrix. It has also been reported that calcium chelators can be potentially used for treating iron overload-related diseases as they help inhibit the influx of iron by regulating the process of TfR1 internalization (Cui et al., 2019). Jing et al. reported that the calcium chelator BAPTA-AM could decrease the extent of iron influx realized into chondrocytes. This helped inhibit iron overload-stimulated ROS accumulation and mitochondrial dysfunction (Jing et al., 2021b). In addition, D-mannose exerted a chondroprotective effect by attenuating the sensitivity of chondrocytes toward ferroptosis and alleviating OA progression. D-mannose protects chondrocytes from ferroptosis by preventing the accumulation of lipid and lipid peroxide. It was also observed that D-mannose enhanced CPT1A-mediated β-oxidation to increase the extent of lipid droplet accumulation realized (Zhou X. et al., 2021).

Subchondral bone integrity and bone remodeling also played important roles in the physiopathology of OA. Iron chelation therapy for abnormal subchondral bone remodeling under iron overload conditions can also be an effective therapeutic strategy. Cornish et al. demonstrated that iron-binding lactoferrin effectively suppressed osteoclast differentiation of monocytes and significantly increased the extent of bone formation in adult mice (Cornish et al., 2004). Iron overload conditions usually resulted in an elevated RANKL/OPG ratio. Hou et al. indicated that the iron-chelating lactoferrin could repair bone density by reducing the RANKL to OPG ratio (Hou J.-m. et al., 2012). It has also been observed that DFO directly suppressed iron-uptake-activated osteoclast formation by negatively regulating the MAPK signaling pathway. The negative effect was not ROS-dependent (Zhang et al., 2019). Some anti-oxidation drugs are being studied to reduce oxidative stress and hinder the apoptosis of osteoblasts that is induced under conditions of iron overload. For example, melatonin dampens the promoting effect of iron overload on the process of osteogenic differentiation dysfunction and senescence by inhibiting the processes of ROS generation and p53/ERK/p38 activation (Yang et al., 2017). Icariin could promote the process of osteoblast survival and reverse the downregulation of the expression of Runx2, alkaline phosphatase (ALP), and osteopontin (OPN) (induced by iron overload), and this effect could be attributed to the protection against mitochondrial membrane potential dysfunction and ROS production (Jing et al., 2019).

**FIGURE 3:**
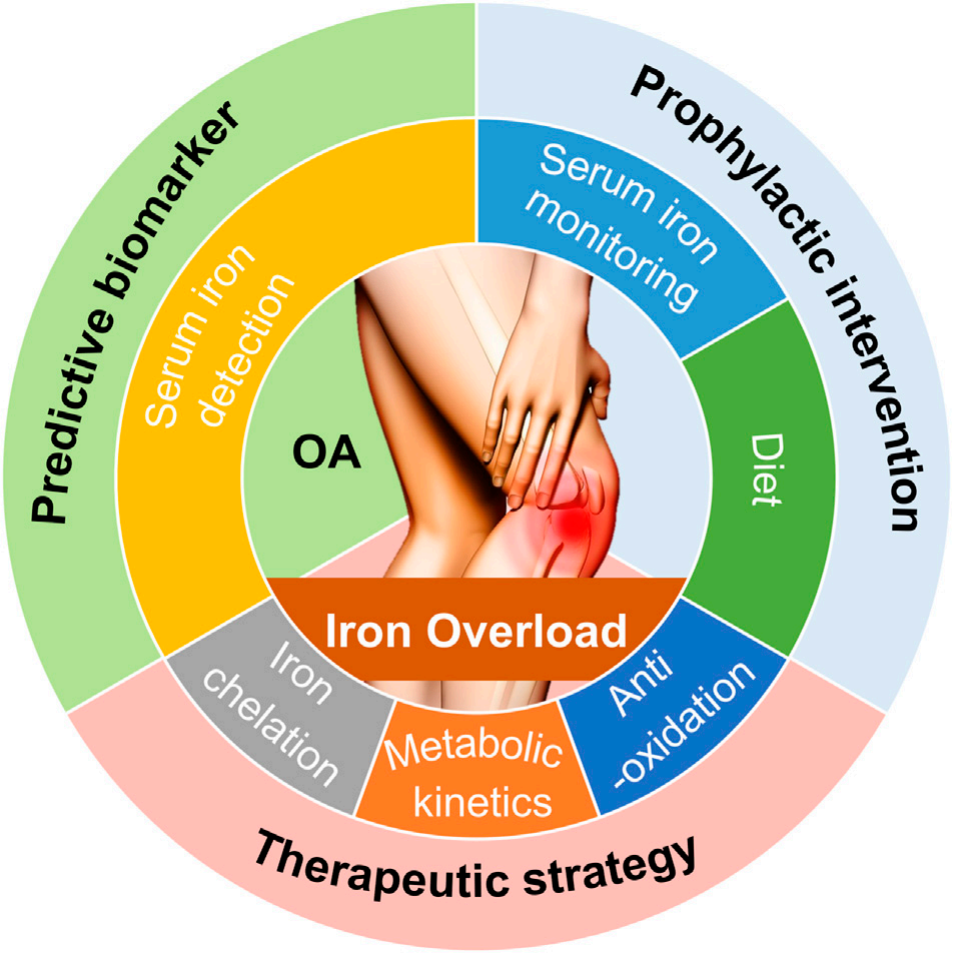
Potential clinical interventions and value associated with iron overload used for predicting, preventing, treating OA. Based on the principles for iron overload-modulated OA pathogenesis that we have discussed, potential clinical interventions and value for OA include predictive biomarker, prophylactic intervention, and therapeutic strategy.

We discuss further literature from the perspective of therapeutic measures in iron metabolic kinetics. Systemic iron homeostasis is maintained by delicately regulating the process of iron acquisition, storage, and export (Bogdan et al., 2016). These processes should be effectively controlled to avoid excess iron-induced detrimental effects. Hepcidin, produced by the liver, is a vital regulator of the process of iron homeostasis. It has been previously reported that the inhibition of hepcidin increases the extent of iron uptake in bone and liver, resulting in increased osteoclast and reduced osteoblast activities (Xu et al., 2017). Jiang et al. reported that hepcidin could also address the problems posed by impaired bone formation caused by FAC treatment by regulating the process of iron absorption (Jiang et al., 2019) (Figure 3; Table 2). Therefore, the development of hepcidin agonists should be studied to better understand the treatment prospects of iron overload-induced OA.

**TABLE 2:** Potential clinical interventions targeting iron overload for the treatment of OA.

Therapeutic targets	Agents	Effects	References
Chondrocytes	Deferoxamine	Inhibit oxidative stress and mitochondrial dysfunction	Jing et al. (2021a)
		Reduce IL-1β-induced matrix-degrading enzymes production	Jing et al. (2020)
	BAPTA-AM	Blunt iron influx through regulating TfR1 internalization	Jing et al. (2021b)
	D-mannose	Protect chondrocytes from ferroptosis by eliminating HIF-2α-dependent lipid peroxide accumulation	Zhou et al. (2021b)
Osteoclasts	Lactoferrin	Suppress osteoclast differentiation via reducing the RANKL/OPG ratio	Hou et al. (2012a)
	Deferoxamine	Inhibit osteoclast formation by negative regulation of MAPK signaling	Zhang et al. (2019)
Osteoblasts	Melatonin	Prevent osteogenic differentiation by inhibiting ROS production and p53/ERK/p38 activation	Yang et al. (2017)
	Icariin	Promote osteoblast survival by preventing mitochondrial membrane potential dysfunction and ROS production	Jing et al. (2019)
Iron metabolic kinetics	Hepcidin	Rescue impaired bone formation caused by FAC treatment via regulating iron absorption	Jiang et al. (2019)

## 5 Prospect

Results from clinical and basic studies reveal that iron overload significantly influences the progression of OA. Results from *in vivo* experiments reveal that an excess of iron is directly associated with the pathological processes associated with the tissues present in the joints in iron-loaded models. In this review, apart from the discussion on the relationship between hereditary blood diseases and iron overload-induced OA, we have also discussed the recent findings reported on aging- and estrogen deficiency-associated OA. We have attempted to understand and uncover the mechanism associated with iron overload. OA is a heterogeneous disorder with different etiologies and is characterized by various subtypes. Further research should be conducted to explore the role of iron overload on the progression of different types of OA. The origin of iron overload and its relation with inflammation, mechanical stress, and energy metabolism should also be studied. It is yet to be ascertained if an increase in the iron level is the only factor that results in pathological arthropathies in patients suffering from OA. Therefore, the influence of other contributing factors such as mechanical loading, altered metabolism, and oxidative stress should also be considered.

Apart from descriptive investigations, cellular and mechanistic explorations are required to help translate the results into strategies for OA treatment. The articular cartage, subchondral bone, and synovial lining of the joint cavity were also studied (Kon et al., 2016; Mathiessen and Conaghan, 2017; Simon and Jackson, 2018). Under conditions of iron overload, excess iron disturbs the process of the anabolic/catabolic metabolism of chondrocytes. The balance between osteoclasts and osteoblasts involved in subchondral bone remodeling was affected under these conditions, and it was observed that iron overload compromises synovial homeostasis coordinated by synovial lining hyperplasia. Infiltration of macrophages and lymphocytes, pannus formation, and fibrosis were also affected under conditions of iron overload (Zhang et al., 2011; Ganz, 2016; Li et al., 2018; Jing et al., 2020). Moreover, OA is characterized by the phenotype of osteophyte formation, cartilage degradation, synovitis, and subchondral bone remodeling (Lieben, 2016). The influence of iron overload on the process of osteophyte formation is unknown and is worthy of further study. Iron overload compromises cellular homeostasis, triggering adverse events (such as oxidative stress, mitochondrial dysfunction, and apoptosis or autophagy), which ultimately results in articular cartilage degeneration, abnormal subchondral bone remodeling, and synovial inflammation. Accumulation of free iron is also a key initiator of ferroptosis, and ferroptosis might be involved in the progression of OA. However, the role of ferroptosis in OA is not well understood. Understanding the process of ferroptosis can potentially open a new area for researchers and help develop a potential target for the treatment of OA.

## 6 Conclusion

OA refers to inflammatory diseases that occur in the joint and surrounding tissues of the human body and are caused by inflammation, degeneration, trauma, or other metabolic factors (Bijlsma et al., 2011). The clinical manifestations of arthritis are joint dysfunction and deformity, which seriously affect the quality of life of patients (Taruc-Uy and Lynch, 2013). Iron overload refers to the accumulation of excess iron in the circulatory system and tissues. This can potentially damage the cells through the generation of peroxide stress. Iron overload can cause pathological changes in various tissues and organs (Calişkan et al., 2011; Yu et al., 2020). The condition of iron overload is frequently associated with the osteoarthritic phenotypes (such as progressive cartilage erosion, altered subchondral bone microarchitecture, and biomechanics, persistent joint inflammation, proliferative synovitis, and synovial pannus) (Heiland et al., 2010; Simão et al., 2019). Evidence from clinical studies and animal models suggests that iron overload is associated with OA (Husar-Memmer et al., 2014; Kiely, 2018; Simão and Cancela, 2021). At present, iron chelators can be used in the field of first-line therapeutic therapy to eliminate iron overload in vital organs, and osteochondral tissue, and synovium (Rao, 2013; Jansová and Šimůnek, 2019). The relationship between iron metabolism and OA development is to be elucidated. Due to the complexity of the molecular mechanism of iron metabolism and the involvement of multi-category cells, there is a lack of information on the mechanism and signal molecules associated with osteochondral and synovium damage attributable to iron overload. Further research needs to be conducted to understand the exact mechanisms resulting in joint damage, and new strategies should be developed to address the problems.

## Glossary

SCD: sickle cell disease
OA: osteoarthritis
HH: hereditary hemochromatosis
HFE: homeostatic iron regulator
HAMP: hepcidin antimicrobial peptide
Tf: transferrin
TfR2: transferrin receptor-2
HJV: hemojuvelin
MMP13: matrix metalloproteinase-13
ADAMTS5: a disintegrin and metalloproteinase with thrombospondin motifs 5
KO: knockout
TNF-α: tumor necrosis factor-α
IL-1β: interleukin-1β
FAC: ferric ammonium citrate
ROS: reactive oxygen species
TfR: transferrin receptor
BMSCs: bone marrow mesenchymal stem cells
RANKL: receptor activator of nuclear factor-kappa B ligand
BMMs: bone marrow-derived macrophages
OPG: osteoprotegerin
DFO: deferoxamine
MAPK: mitogen-activated protein kinase
Runx2: runt-related transcription factor 2
DMT1: divalent metal transporter 1
PI3K: phosphatidylinositol 3-kinase
JAK: janus kinase
STAT3: signal transducer and activator of transcription 3
FLSs: fibroblast-like synoviocytes
RA: rheumatoid arthritis
ALP: alkaline phosphatase
OPN: osteopontin.
